# Computational fluid dynamic analysis of upper airway procedures in equine larynges

**DOI:** 10.3389/fvets.2023.1139398

**Published:** 2023-04-17

**Authors:** Michelle L. Tucker, David G. Wilson, Donald J. Bergstrom, James L. Carmalt

**Affiliations:** ^1^Veterinary Clinical Sciences, College of Veterinary Medicine, Purdue University, West Lafayette, IN, United States; ^2^Department of Large Animal Clinical Sciences, Western College of Veterinary Medicine, University of Saskatchewan, Saskatoon, SK, Canada; ^3^Department of Mechanical Engineering, University of Saskatchewan, Saskatoon, SK, Canada

**Keywords:** equine, computational fluid dynamics modeling (CFD), laryngeal hemiplegia, recurrent laryngeal neuropathy (RLN), laryngoplasty, partial arytenoidectomy, airway resistance, airway mechanics

## Abstract

**Introduction:**

Computational fluid dynamics (CFD) has proven useful in the planning of upper airway surgery in humans, where it is used to anticipate the influence of the surgical procedures on post-operative airflow. This technology has only been reported twice in an equine model, with a limited scope of airflow mechanics situations examined. The reported study sought to widen this application to the variety of procedures used to treat equine recurrent laryngeal neuropathy (RLN). The first objective of this study was to generate a CFD model of an *ex-vivo* box model of ten different equine larynges replicating RLN and four therapeutic surgeries to compare the calculated impedance between these procedures for each larynx. The second objective was to determine the accuracy between a CFD model and measured airflow characteristics in equine larynges. The last objective was to explore the anatomic distribution of changes in pressure, velocity, and turbulent kinetic energy associated with the disease (RLN) and each surgical procedure performed.

**Methods:**

Ten equine cadaveric larynges underwent inhalation airflow testing in an instrumented box while undergoing a concurrent computed tomographic (CT) exam. The pressure upstream and downstream (outlet) were measured simultaneously. CT image segmentation was performed to generate stereolithography files, which underwent CFD analysis using the experimentally measured outlet pressure. The ranked procedural order and calculated laryngeal impedance were compared to the experimentally obtained values.

**Results and discussion:**

The CFD model agreed with the measured results in predicting the procedure resulting in the lowest post-operative impedance in 9/10 larynges. Numerically, the CFD calculated laryngeal impedance was approximately 0.7 times that of the measured calculation. Low pressure and high velocity were observed around regions of tissue protrusion within the lumen of the larynx. RLN, the corniculectomy and partial arytenoidectomy surgical procedures exhibited low pressure troughs and high velocity peaks compared to the laryngoplasty and combined laryngoplasty/corniculectomy procedures. CFD modeling of the equine larynx reliably calculated the lowest impedance of the different surgical procedures. Future development of the CFD technique to this application may improve numerical accuracy and is recommended prior to consideration for use in patients.

## 1. Introduction

Partial upper airway obstruction as a result of equine recurrent laryngeal neuropathy (RLN) results in decreased performance and represents a complex surgical problem for the performance horse population. While there have been a large number of studies looking at the laryngoplasty (1, 2) and partial arytenoidectomy (3) surgical techniques and their effects on performance, there are still many suboptimal postoperative outcomes. *Ex vivo* models have contributed significantly to the refinement of these procedures, and further exploration of how laryngeal manipulation influences airflow development.

In particular, the equine “laryngeal box” flow system has explored the influence of various surgeries on overall airflow through the larynx. It allows for a controlled environment in which the effect of laryngoplasty (4–8), cricoarytenoid joint stabilization (9), vocal cordopexy (5), vocal cordectomy (10), partial arytenoidectomy (8), and the arytenoid corniculectomy (8) surgeries have been studied. This system was used to prove that the translaryngeal impedance was related to prosthesis shortening and arytenoid abduction (7) and demonstrated the mechanical advantage of cricoarytenoid joint stabilization (9). Despite this, a recurring theme of “individual variability” was observed across the studies. While the box system continues to be used as a preliminary testing mechanism for new surgical ideas, it does not completely capture the intricacies of laryngeal mechanics nor the outcomes observed in individual clinical patients.

The benefit of combining computational fluid dynamics (CFD) to clinical knowledge is exemplified within human respiratory surgery. CFD studies have led to a greater understanding of the relationship between the geometry of the upper airway and obstructive airway disorders (11–16), such as the pharyngeal length and increasing degrees of collapse (15). This has allowed surgeons to make better decisions regarding treatment type and expected procedure efficacy for complicated problems such as obstructive sleep apnea (11–15, 17) syndrome and nasal septal deviation (16); even going so far as to develop individualized patient treatments (16, 17).

Similarly, an equine upper airway CFD model that replicated the anatomy from nares to trachea (18) has been reported. The model replicated the inhalation and exhalation cycles of an exercising horse with the anticipated air velocity and volumetric flow rates, demonstrating validity in the application of the equine patient (18, 19). A follow up study demonstrated the use of the model to investigate equine surgeries and found that 88% arytenoid abduction was an acceptable level to potentially improve performance in horses with arytenoid cartilage collapse (20). CFD has also been employed in the optimization of an equine nasal metabolic mask design to ensure that the mask would function as desired without significantly increased airway impedance (21). While CFD provides more specific and comprehensive information about the fluid mechanics in the equine subject, its continued validation and advancement are needed in this field to widen its applications.

The objectives of this study were to generate a CFD model of ten different equine larynges replicating RLN and four therapeutic surgeries based on an *ex vivo* box model to compare within-larynx impedances. Secondly, to compare the calculated and measured impedance values as a measure of the numerical accuracy of the CFD model. Thirdly, to examine the effect of RLN and four different surgical interventions on anatomical distributions of pressure, velocity and turbulent kinetic energy. The first hypothesis was that the CFD model would be able to accurately predict the surgical procedure generating the lowest impedance compared to the experimental data for a specific larynx. The second was that negative pressure troughs, velocity peaks, and turbulence spikes would occur in the disease state and in those surgical procedures that created the highest impedance and generated the most collapse of the laryngeal tissue, such as the corniculectomy and partial arytenoidectomy.

## 2. Materials and methods

### 2.1. Experimental data and image collection

Ten larynges were collected from equine cadavers with no reported history of upper airway disease as previously described (8). Ages of subjects ranged from 4 to 29 years, with six Quarter Horses, one Thoroughbred, one Canadian Warmblood, one American Paint and one horse of unknown breed included. Subjects were euthanized for given reasons of colic (1), degenerative joint disease (3), septic arthritis (2), behavior (1), severe osteochondral fragments in the stifle (1) and reasons not specified by the clinician (2). Each larynx was collected within 2 h of euthanasia along with the esophagus and surrounding tissues and wrapped in saline-soaked gauze. They were placed in a freezer at −20°C until the day before testing. Thawing occurred over 20 h by placing the laryngeal specimens in a room at 23°C. Just prior to testing, the esophagus, excess tracheal rings, and remaining extrinsic musculature were removed from each specimen. Four to five tracheal rings were left *in-situ* to allow overlap with a polyvinyl chloride adapter for insertion into an instrumented box. The adapter facilitated attachment of each laryngeal specimen to the box outlet which was 5.08 cm in diameter. Each trachea was matched to a corresponding adapter of either 2.54 or 3.81 cm in diameter and fixed with plastic cable ties to create a seal (7). The epiglottis was anchored to foam in the bottom of the box to prevent dorsal movement and retroversion into the airflow during testing.

**Figure 1:**
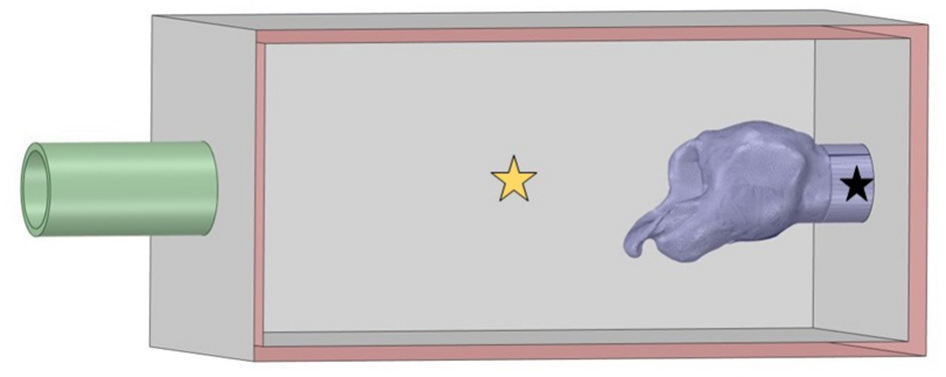
Larynx in box geometry. The inlet on the left was defined as atmospheric pressure and flow proceeded into the box through the larynx to exit at the pipe outlet defined as the measured negative tracheal pressure. Pharyngeal pressure was measured at the yellow star and tracheal pressure at the location denoted by the black star.

Two pressure transducers (Model P55D, Validyne Engineering, Los Angeles, CA) were used to measure the pressure halfway along the distance of the box and at the tracheal PVC adapter at 100 Hz each. An orifice plate (ISO standard 5,167) was used 95 cm downstream from the box outlet to measure the flow rate of air through the trachea using an additional transducer (Model DP103-14, Validyne Engineering, Los Angeles, CA) (22). The flow rate was recorded for a minimum of 30 s using an analog-to-USB adapter (USB-6221 National Instruments, Austin, TX) for each laryngeal trial and monitored using a commercial software (LabView, National Instruments, Austin, TX). Pressure within the trachea and box were recorded, along with flow rate, as previously described (8). Placement of the pressure tranducers measuring the “pharyngeal” pressure and tracheal pressure can be seen in Figure 1. Concurrent digital video recording was performed using a USB camera (Logitech, C920 Web camera, Newark, CA) during the simultaneous computed tomographic (CT) exam. CT exams were performed at 200 mAs, 120 kVp with a 0.64 mm pitch with 1 mm slice thickness at a scan speed of 1 mm/s using helical scans using a Toshiba Aquilon One (Canon Medical Systems, Markham, ON, CA).

Each larynx underwent the same testing sequence, resulting in 5 different trials per larynx which were performed in immediate succession, within less than an hour total time. All procedures were performed by the primary author following multiple practice sessions to ensure consistency in technique and efficiency in the workflow. First, left RLN was imitated by performing a right laryngoplasty with a single piece of #5 Ethibond suture (Ethibond Excel, Ethicon US LLC, Bridgewater, NJ). The cricoid cartilage bite was performed from ventral to dorsal, approximately 10 mm rostral to the caudal border and medial to any palpable notches within 10 mm from the sagittal ridge. The arytenoid bite was placed into the spine of the muscular process in a dorsomedial to ventrolateral direction. Tension was placed to ensure maximal abduction of the right arytenoid cartilage and a surgeon's throw was performed followed by three square throws, ensuring that tension was maintained. The larynx was tested with the right arytenoid cartilage abducted and the left arytenoid cartilage allowed to collapse. Once adequate data was collected, a left laryngoplasty with ipsilateral ventriculocordectomy (LLP) was performed using a roaring burr to exteriorize the left ventricular mucosa for excision (23). The remaining mucosa was glued using cyanoacrylate glue to minimize excessive tissue collapse and replicate the postoperative mucosal healing. Following the airflow trial, a left arytenoid corniculectomy was performed by excising the corniculate process of the left arytenoid (LLPCOR). Once this was tested, the left laryngoplasty suture was cut and removed from the larynx, to imitate a simple corniculectomy with ipsilateral ventriculocordectomy procedure (COR). This was tested and then a partial arytenoidectomy (PA) procedure was performed by removing the body of the left arytenoid cartilage (24), followed by a final round of testing. All flow settings were monitored and adjusted to a target tracheal pressure of −4,300 Pa per run with as high a flow possible.

### 2.2. Segmentation

The DICOM files from the CT scans were imported into Fiji (ImageJ, National Institutes of Health, Bethesda, MD) for segmentation. Automatic segmentation was performed using the 3D viewer plugin, examined slice-by-slice, then exported as binary stereolithography file (.stl). These were then imported into Ansys^®^ SpaceClaim (Ansys 2021 R1, ANSYS, Inc., Canonsburg, PA) to remove plastic cable ties and artifact elements from the metal anchor used to pin the epiglottis and any unattached CT artifacts from the laryngeal geometry. The CT resulted in mirror image geometry, which was flipped and imported as a stereolithography file (.stl) into MeshLab (MeshLab version 2020.12, Visual Computing Lab, CNR-ISTI, Pisa, Italy). The non-manifold vertex and face filters were applied to clean the mesh, and the HC Laplacian smooth function (25) was applied for twenty iterations until the geometry appeared smooth (21). The remesh filter was applied to minimize the file size associated with the mesh. The mesh was exported as an .stl file which was reintroduced into SpaceClaim. The sharp edge detection tool and mesh editing tools were used to clean the geometry further until the faceted body could be converted into a solid body. This was adjoined with the computer-aided design (CAD) geometry for the box and inlet and outlet PVC pipes to replicate the experimental system. The inlet and outlet were labeled in SpaceClaim and the geometry subsequently imported into Ansys^®^ Fluent (Ansys 2021 R1 build 10179, ANSYS, Inc., Canonsburg, PA) for meshing and computation. This is shown in Figure 1.

A mesh independence study was performed to determine optimal mesh size for the variables of interest, namely the calculated within-box pressure, calculated airflow rate and laryngeal impedance. The resulting ideal mesh size was 11 million cells or greater. A local size element of 0.25 mm was used for the surface mesh around the larynx, while 0.5–15 mm was used throughout the box and pipe with a growth rate of 1.2. Smooth transition boundary layers were applied with 5-layer depth. A polyhedral mesh was generated using the meshing tool under these conditions for each procedure for each larynx.

### 2.3. Numerical model

The calculations were performed in Fluent using the finite volume method. Air was assumed to be incompressible with density 1.225 kg/m^3^ and viscosity 1.789 × 10^−5^ kg/(ms). Rigid walls were assumed with dry conditions and no respiratory mucus as has been previously reported (18, 21). This circumvented the need for a complex fluid-fluid interaction model. A realizable κ-ε turbulence model was solved using the Semi-Implicit Method for Pressure Linked Equations (SIMPLE) method for the Reynolds-Averaged Navier Stokes (RANS) equations. A standard wall function was implemented (26).

#### 2.3.1. Boundary conditions

Each case was set up as a pressure differential problem. At the inlet of the pipe pressure was set at 0 Pa. For the outlet pipe, the opening pressure was defined as the measured tracheal pressure for that treatment/larynx, which was on average −4,300 Pa. Zero velocity was assumed along the walls. The walls were defined as “no slip” with a smooth transitional boundary mesh. Calculations were performed on a 16-core, 4.30 GHz AMD Ryzen Threadripper processer (Advanced Micro Devices, Santa Clara, CA) with 128 GB RAM.

#### 2.3.2. Mesh independence study

**Figure 2:**
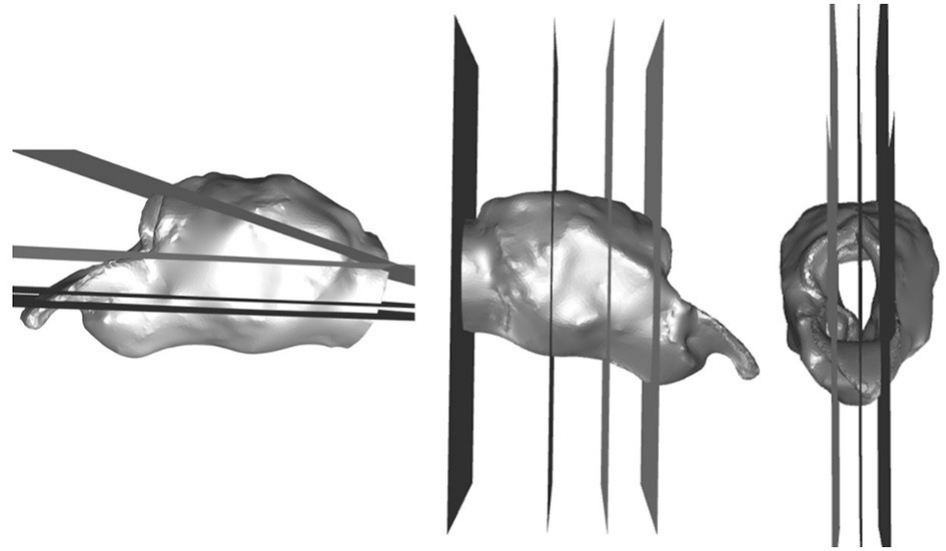
Planes generated for the independent mesh study and qualitative analysis. Planes in three orthogonal directions generated in Fluent to analyze the flow characteristics in anatomical and geometrical areas of interest.

The mesh independence study was conducted using the RLN state for the fifth larynx, which was anticipated to exhibit high resistance with resultant complex flows. Planes of interest were generated parallel to the axial, sagittal and transverse planes to capture the average and minimum pressure for each plane, with mesh sizes of 1.5, 3, 5.8, 11.5, and 17.9 × 10^6^ cells to determine the change between each of the increases in size and the trend of the solutions. The planes of interest are shown in Figure 2. The variables of greatest clinical interest were pressure, flow rate, and impedance. These changed in clinically insignificant proportions between the 11 and 17 million cell models (4–5, 0.004, and 1.4% respectively). The computer was unable to proceed with calculations above 17 million mesh elements. The 11 million cell model took less than an hour to run 1,000 iterations in most instances. This was sufficient to achieve stable residual reduction of more than 10^−2^ in most cases.

### 2.4. Data analysis

For each of the five tests for each larynx the computational result was analyzed for the given impedance across each larynx. This was calculated by taking the area-averaged box pressure just upstream from the larynx and subtracting this from the area-averaged outlet pressure divided by the calculated air flow rate, as shown in the equation below.


$$\begin{array}{l} Impedance\;=\;\frac{P_{Box}-P_{Trachea}}{Flow\;Rate} \end{array}$$

A mixed effects model was performed examining the effect of larynx and procedure on the CFD-derived impedance using larynx number as a subject-level effect. Subsequent pairwise comparisons were then performed using Tukey-Kramer between procedures. This was performed in SAS 9.4 (SAS Institute Inc., Cary, NC) with significance set at *p* < 0.05.

Qualitative analysis of the velocity, pressure, and turbulent kinetic energy production was performed by the primary author for each procedure within larynx by creating planes of interest as for the mesh independence study. Three sagittal planes, four transverse, and four cross-sectional planes were generated to examine the flow characteristics within the larynges. Each of these planes was cropped to focus on the laryngeal region for comparison. The sagittal planes were generated at midline, then a parasagittal left and parasagittal right to examine the effects just inside the walls on the left and right side of the larynx. Transverse planes were generated to divide the larynx in half, then mid-saccule, and an additional dorsal and ventral plane halfway between the limits of the lumen and the existing planes, and slightly angled to incorporate the trachea to approximately the level of the first tracheal ring. Cross-sectional planes were generated at the laryngeal opening, mid-saccule, and caudal to the saccules and a fourth plane within the trachea.

For each larynx, the pressure, velocity and turbulent kinetic energy color scales were standardized across procedures to determine the influence of each procedure within the larynx. When an area of particularly low pressure or high velocity was observed in one plane, the region was examined within the other orthogonal planes to determine whether the trend observed was verifiable. Images were evaluated for areas of exceptionally low pressure (<-5.2 kPa), high velocity (>90 m/s), and high turbulent kinetic energy (>250 m^2^/s^2^). These were denoted by the presence of yellow (approximately −5 to −6 kPa) or green (approximately −6 to −10 kPa) colors on the pressure images, green (approximately 55–85 m/s), yellow (approximately 85–100 m/s) or orange (approximately 100–110 m/s) colors on the velocity images and light blue or green (230–430 m^2^/s^2^) in the images representing turbulent kinetic energy. The anatomical locations associated with each were noted where possible, and the localization and presence of that region of low pressure, high velocity or high turbulent kinetic energy was confirmed using the orthogonal views associated with each image. The various procedures were compared within larynx and then between larynges. When characteristics were seen that warranted even closer examination, the three-dimensional streamline (velocity, turbulent kinetic energy) or wall renderings (pressure) were referenced to further characterize the flow effects.

## 3. Results

### 3.1. Quantitative results

**Figure 3:**
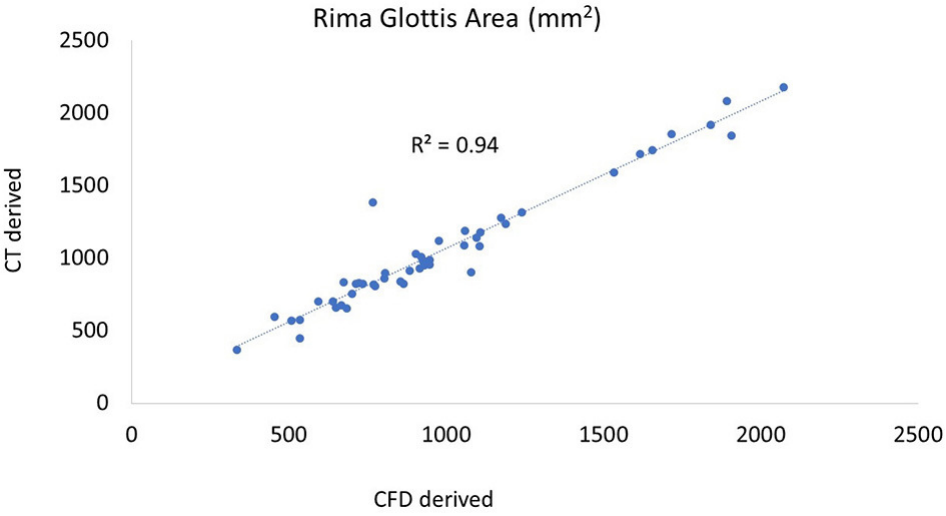
Plot of CT vs. CFD model derived rima glottis area. The CT-derived area was taken from the coronal plane, the first complete area of tissue. A similar plane was obtained in the 3D geometry in SpaceClaim following processing and the cross-sectional area measured.

The rima glottis cross-sectional areas were compared between CT measurements and the final geometry for all procedures across all larynges and had a Pearson correlation coefficient of 0.97 which would indicate that the methods used to produce the three-dimensional models here were effective in appropriately capturing the geometry for numerical analysis (Figure 3).

For 7 out of 10 larynges, the CFD model determined that the same surgical procedure generated the lowest impedance that was reported experimentally. The procedure of choice by larynx along with the experimentally measured and CFD-calculated impedance are listed in Table 1. For 2 of the larynges, there were two procedures where the CFD and experimental “procedure of choice” were different, but per the impedance values, the difference is 0.02 or less. For larynx 3, the computational model and experimental measurement resulted in different procedures of lowest impedance, but computationally there were two procedures that were close in impedance.

**Table 1:** Procedure of lowest impedance for each larynx.

		**Impedance (kPa** ^*^ **s/L)**	
**Larynx #**	**Surgical procedure**	**CFD**	**EXP**
**1**	**LLP**	0.177	0.195
**2** ^*^	**LLP**	0.026	0.024
**2** ^*^	**LLPCOR**	0.026	0.026
**3** ^**^	**LLPCOR**	0.109	0.169
**3** ^**^	**PA**	0.107	0.201
**4**	**LLP**	0.030	0.041
**5**	**LLPCOR**	0.058	0.084
**6**	**LLP**	0.039	0.048
**7**	**LLP**	0.055	0.080
**8** ^*^	**LLP**	0.067	0.099
**8** ^*^	**LLPCOR**	0.065	0.118
**9**	**LLPCOR**	0.051	0.093
**10**	**LLP**	0.038	0.045

EXP-experimentally measured laryngeal impedance. ^*^For larynges 2 and 8 there were two procedures with lowest impedances that differed by <0.02. ^**^For larynx 3, the CFD and EXP impedance did not agree for the “best” procedure so both are reported for comparison.

**Figure 4:**
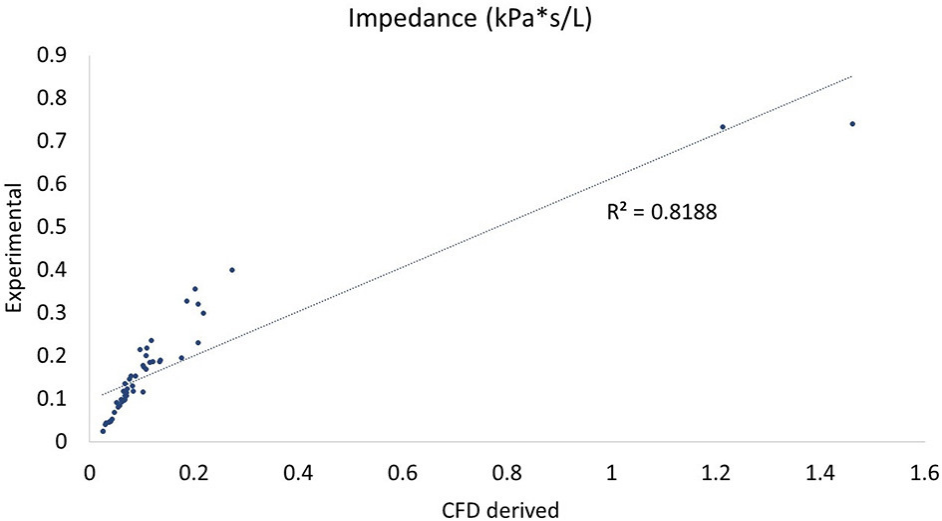
Plot of CT vs. CFD model derived impedance. Plot of impedance in kPa*s/L, flow rate with correlation coefficient (r^2^). There were two outlying data points with unusually low reported flow rates which can be seen to the far right of the impedance plot.

**Figure 5:**
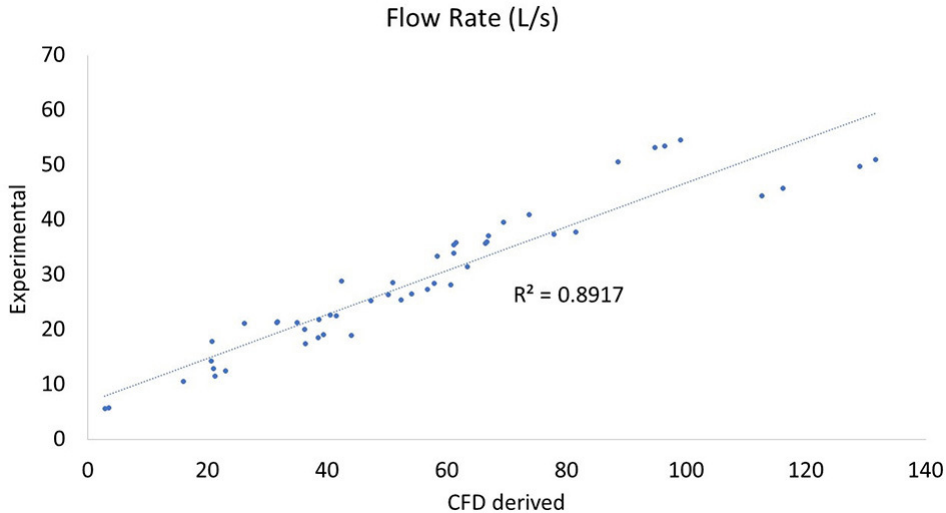
Plot of CT vs. CFD model derived flow rate. Plot of flow rate in L/s with the correlation coefficient (r^2^). There were two outlying data points with unusually low reported flow rates which can be seen at the bottom of the plot and correspond to the two outlying points in the impedance plot (Figure 4).

**Figure 6:**
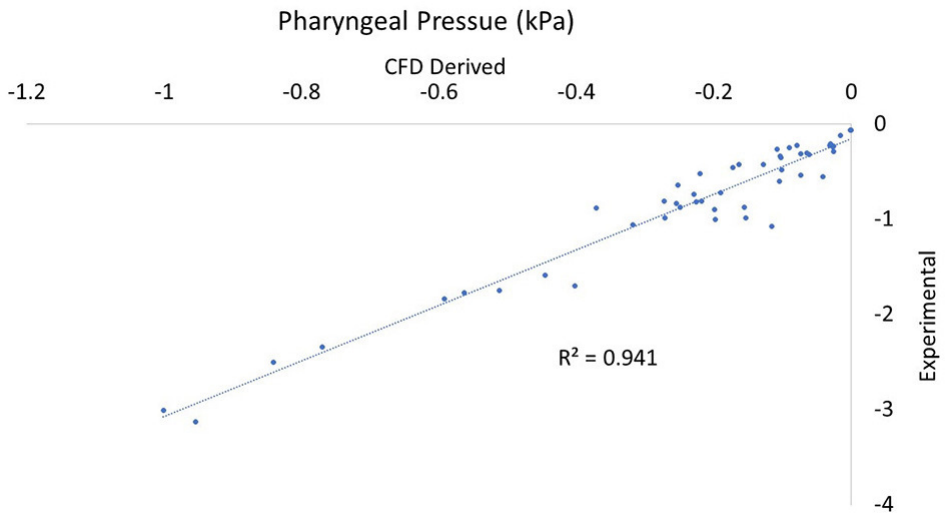
Plot of CT vs. CFD model derived pharyngeal pressure. Plot of impedance pharyngeal pressure in kPa with the correlation coefficient (r^2^). Note that all values are negative as they are relative to the pressure in the room.

In comparing experimental vs. computed values for flow rate, pharyngeal pressure, and impedance, plots were performed (Figures 4–6). The calculated values may be found in Table 2. The mixed effects statistical model was constructed accounting for larynx and procedure and examining the effects on CFD calculated impedance. RLN was found to be significantly different from the surgical procedures, LLP was significantly different from COR but there were no other significant differences between surgical procedures. The resultant *p*-values from the Tukey-Kramer pairwise comparisons with significant differences are shown in Table 3.

**Table 2:** CFD-reported values reported by procedure for average and range of airflow (L/s) and pharyngeal pressure (kPa).

**Procedure**	**Pharyngeal pressure (kPa)**	**Air flow (L/s)**	Impedance (kPa^*^s/L)
**RLN**	−0.073 (−0.001 to −0.190)	31.1 (2.87–58.4)	0.280 (0.071–1.46)
**LLP**	−0.404 (−0.042 to −0.954)	76.7 (26.1–131.7)	0.068 (0.026–0.177)
**LLPCOR**	−0.406 (−0.026 to −1.00)	76.3 (20.7–129.0)	0.070 (0.026–0.22)
**COR**	−0.084 (−0.001 to −0.402)	43.4 (3.49–81.5)	0.210 (0.047–1.21)
**PA**	−0.156 (−0.312 to −0.272)	49.1 (23.1–66.5)	0.094 (0.061–0.19)

Values reported as mean (range).

**Table 3:** Resultant p-values between procedures from the mixed effects statistical model by procedure.

	**PA**	**COR**	**LLPCOR**	**LLP**	**RLN**
**RLN**	<0.0001^*^	0.0054^*^	<0.0001^*^	<0.0001^*^	**—**
**LLP**	0.4114	0.0169^*^	0.9997	—	
**LLPCOR**	0.5236	0.270	—		
**COR**	0.5558	—			
**PA**	—				

^*^Significantly different impedances between these two procedures with *p* < 0.05.

### 3.2. Qualitative results

**Figure 7:**
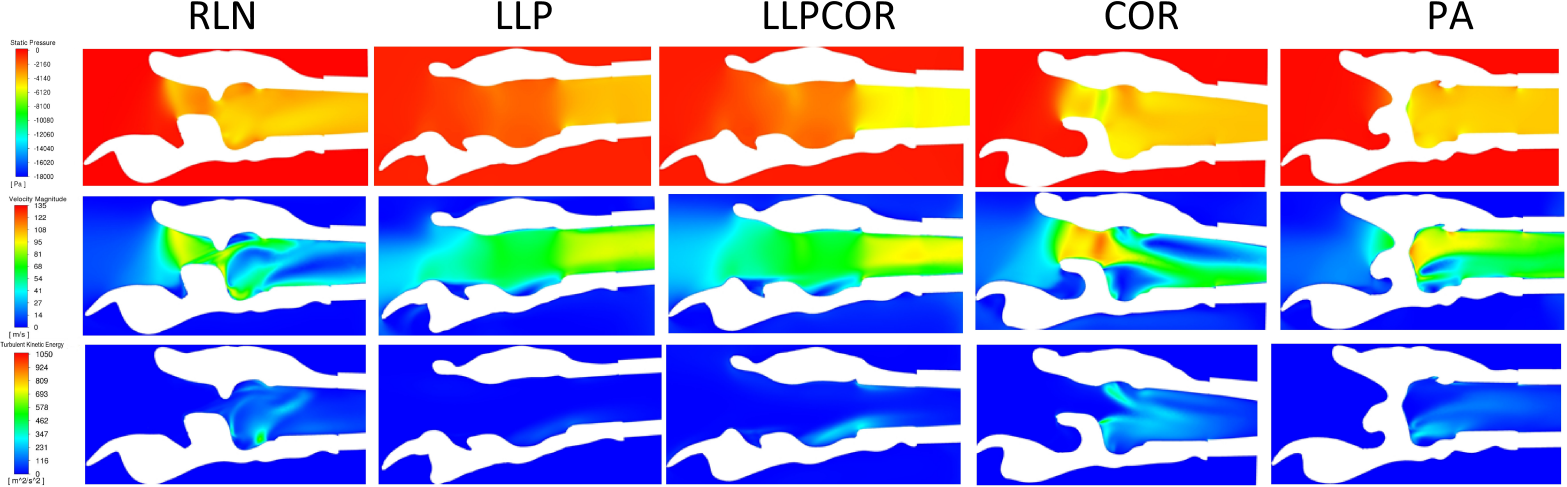
Example of the sagittal sections for larynx 10, showing the velocity **(top)**, pressure **(middle)** and turbulent kinetic energy **(bottom)**. The white region in the plane sections represents the laryngeal tissue, and the colored regions represent the region of airflow. Sections such as this were created for each larynx as described and then examined for the changes of pressure, velocity, and turbulent kinetic energy as they related to different anatomical portions of the larynx.

All of the slices from each procedure for each larynx were placed side-by side and compared as reported in the Table 4. Characteristics across larynges are reported as follows for pressure, velocity and turbulent kinetic energy. An example of the sagittal sections for these values for larynx 10 can be found in Figure 7.

**Table 4:** Summary of qualitative results by larynx.

**Larynx #**	**Pressure**	**Velocity**	**Turbulent Kinetic Energy**
**1**	LLP: Right arytenoid; mid-ventricle (green)	RLN, COR: Jet formation, right side higher than left (green)	LLP, LLPCOR, PA: Increased caudal larynx
	LLPCOR, PA: Peak left arytenoid wall (green)	LLP, LLPCOR, PA: Increased velocity dorsally (yellow)	
**2**	LLP, LLPCOR: Uniform (orange)	RLN: Mid-ventricle (yellow)	RLN, COR, PA: Increased
	COR, PA: High pressure inside left wall (green)	LLP, LLPCOR: Uniform (green)	PA: Most, caudal portion
		COR, PA: Left dorsal (orange)	
**3**	LLP, LLPCOR: Left dorsal arytenoid (green)	LLP: Increased more caudal, dorsal left arytenoid wall (yellow)	LLP, LLPCOR: Caudal to the saccules
	RLN, COR, PA: Localized to the ventricles (green)	All procedures red at the ventricles	COR: Left dorsal
			PA on left
**4**	RLN, PA: Ventricles (yellow)	RLN, PA: Flow separation from wall (yellow)	COR, PA: Downstream from ventricles
	LLP, LLPCOR: Uniform (orange)	LLP, LLPCOR: Uniform (green)	
	COR: Peak inside left arytenoid (green)	COR: Highest with caudal narrowing (orange)	
**5**	LLP: Peak inside left arytenoid (green)	LLP, LLPCOR: High caudal and inner left arytenoid (yellow)	RLN, LLP LLPCOR: Caudal to ventricles
	LLPCOR: Inside aryepiglottic fold (green)	COR: Rostral ventricles (orange)	PA trial data lost
	COR: Rima glottis, left side (green)	PA trial data lost	
	PA trial data lost		
**6**	RLN, COR, PA: Inside the left arytenoid (green)	RLN: Mid-ventricles (yellow)	RLN, COR: Ventricles
	LLP, LLPCOR: Uniform (orange)	COR :along ventricles (yellow)	PA: Caudal
		LLP, LLPCOR, PA: Uniform (green)	
**7**	LLP, LLPCOR: Low at ventricles(green)	LLPCOR: Coanda effect transverse section (right)	Low turbulence all
	COR: Low inside the left arytenoid (green)	COR: Mid-ventricle, dorsally, narrowing caudally (orange)	
		All: Dorsal flow (green)	
**8**	LLPCOR, COR: Dorsal rima glottis (yellow)	LLPCOR, COR: Increased dorsally (green)	LLP, LLPCOR: Increased caudal to ventricles
		LLP: Increased dorsally left arytenoid (green)	
**9**	LLP, LLPCOR: Left dorsal arytenoid (green)	LLPCOR, COR, PA: Mid-ventricles (orange)	LLP, COR, PA: Caudal to ventricles
	COR: Ventral, caudal to ventricles (green)	RLN, LLP: Dorsal flow (green)	
	LLPCOR, PA: Increased dorsally (green)		
**10**	LLP/LLPCOR: Uniform (orange)	RLN, COR, PA: Mid-ventricle, caudal (orange)	RLN, COR, PA: High dorsal and ventral
	RLN: Inside left arytenoid (yellow)	LLP/LLPCOR: Distribution throughout (green)	LLP/LLPCOR: Low, uniform
	COR: Inside left arytenoid (green)		

#### 3.2.1. Pressure

**Figure 8:**
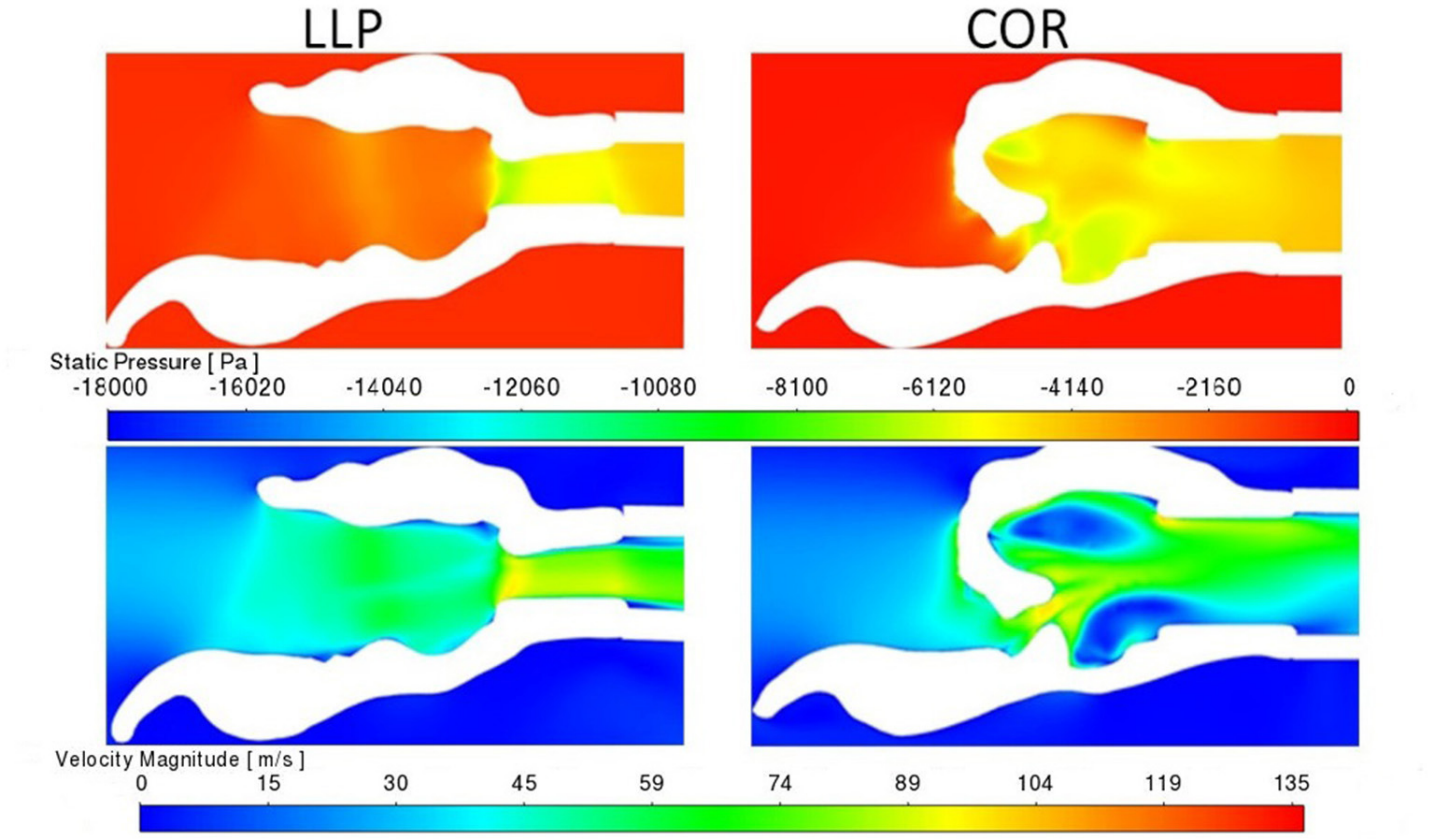
LLP and COR sections inside the left arytenoid cartilage for pressure and velocity for larynx 4. The white region in the plane sections represents the laryngeal tissue, and the colored regions represent the region of airflow. A more gradual transition in pressure and velocity can be observed in the LLP section while the COR section reflects a more obstructive conformation where an abrupt change in pressure and velocity are present.

Pressure for the LLP and LLPCOR procedures was uniformly distributed across the rima glottis opening and from that opening to the level of the trachea, as demonstrated in Figure 8. This was consistent across all larynges. RLN, COR, and PA demonstrated a more abrupt change in pressure associated with the rapid change from a large to small luminal diameter specifically at the level of the rima glottis.

#### 3.2.2. Velocity

**Figure 9:**
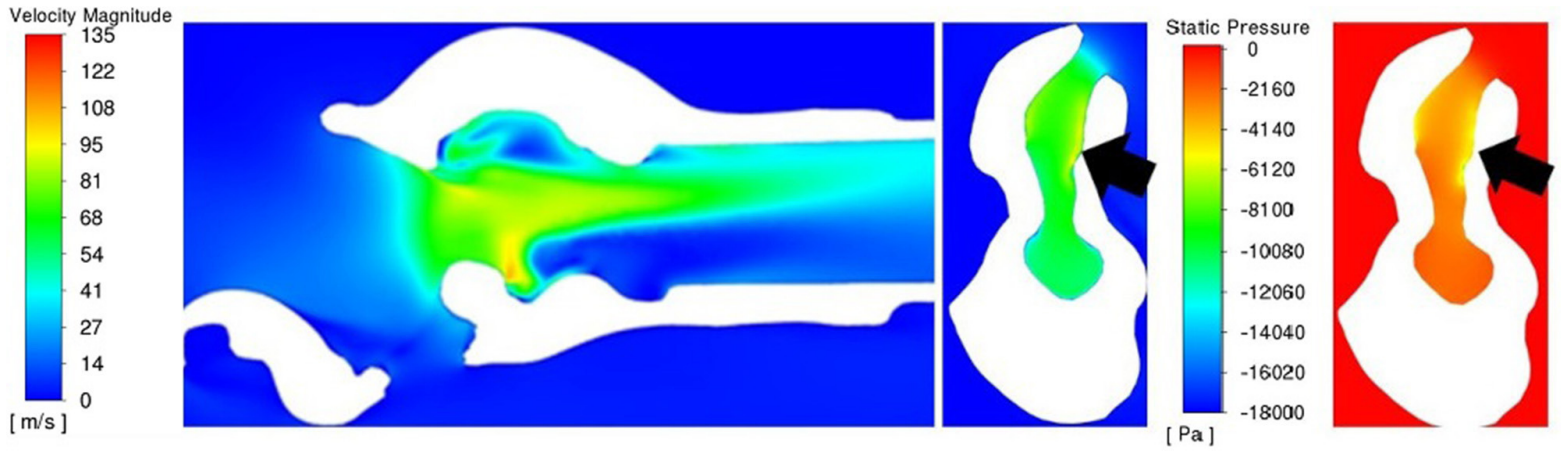
Sagittal velocity section **(left)** and cross-sectional rima glottis section for PA with velocity **(middle)** and pressure **(right)** for larynx 3. Of particular note is the ventral obstruction in the sagittal image and the region of lower pressure and high velocity inside of the left arytenoid cartilage (on the right side of the cross-sectional images, denoted by the black arrows).

A uniformly increased velocity through the lumen was observed for the LLP and LLPCOR procedures, while a higher peak velocity was observed for the RLN, COR and PA procedures. Figure 8 demonstrates the difference between these groupings with relevant cross-sections from the LLP and COR states. Occasional areas of localized negative pressure and high velocity were noted with particular laryngeal geometries, as shown in Figure 9.

#### 3.2.3. Turbulent kinetic energy

**Figure 10:**
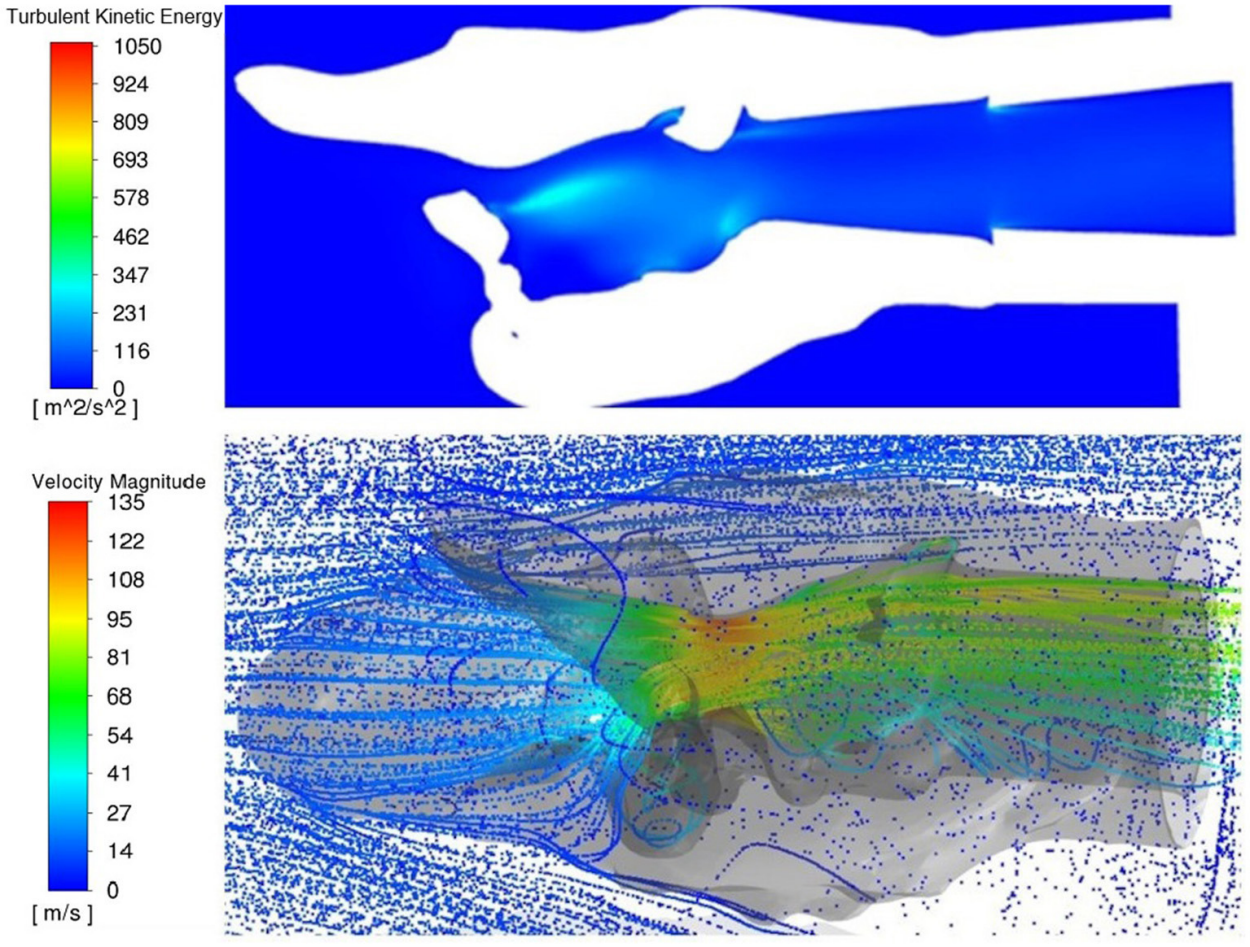
Dorsal transverse section of PA and three-dimensional rendering of the larynx 10, top view. Both images represent a top perspective of the same larynx showing increased turbulent kinetic energy behind tissue obstruction within the larynx and the three-dimensional rendering showing eddy formation in the same region behind the obstruction.

Increased turbulent kinetic energy was frequently observed downstream from tissue protrusions whether from the ventricles and vocal folds, mucosal edge collapse or left arytenoid collapse, especially perpendicular to the plane of flow (Figure 10). Localized regions of increased turbulent kinetic energy were not always observed with more obstructive procedures. Larynges 3 and 5 had greater turbulence in the rostral portions of the LLP and LLPCOR procedures compared to their cohorts.

## 4. Discussion

The CFD model generated in this study agreed with the experimental data in predicting the surgical procedure with the lowest impedance in 9 out of 10 larynges. The statistical findings for this study in regards to procedures differed from the previous study (27) (focused solely on cross-sectional area) in that the CFD results indicated a significant difference between LLPCOR and PA vs. RLN. Additionally, the LLP vs. COR procedures were found to be significantly different for the CFD impedance results, while a significant difference was not found between PA vs. LLP and COR. Thus, a better-fitting turbulence model could be employed in future applications to achieve greater numerical accuracy prior to clinical application. However, it is important to consider that there has been significant variability in reported translaryngeal impedances across studies (4–10, 27), so the consistency of the box model may not be sufficient. This CFD model provided useful information about which surgical procedure would be expected to have the greatest influence on airflow impedance; anatomically it demonstrated where airflow mechanics (low pressure troughs) may contribute to collapse.

Within the CFD model, air was observed to circulate around the larynx within the box, which was expected from previous experiences with the laryngeal box system. This is obviously not reflective of the airflow within an equine patient, wherein air is funneled into the larynx by the pharynx and represents an area of potential improvement. Inclusion of more components of the upper airway would establish a more anatomically-realistic inlet boundary conditions for the CFD model, given that in the live animal, air travels through the nostrils, around the nasal turbinates and ethmoids and then enters the pharynx, which is an area of a high level of mixing and recirculation (19). This presents a very different computational problem compared to the experimental scenario replicated here but may also improve the accuracy of the CFD model in representing an actual patient. Isolating the larynx in this study afforded a controlled mechanism to compare surgical procedures and to examine the accuracy of the CFD model as it pertained to the *ex vivo* (box system) evaluation of airflow mechanics.

It is not entirely clear why larynx 3 had such a disparity between the measured and computational results but it did have distinct collapse features. This larynx was unique in that both of the ventral corniculate processes of the arytenoids collapsed along with the aryepiglottic folds. This level of flaccidity has been observed in clinical patients, so this result is not unlike some of the cases that may be encountered in clinical practice (28). In these cases, the cartilage biomechanics may be the dominant variable in the level of collapse observed. Another larynx (5) in this study exhibited a similar shape of aryepiglottic fold and corniculate collapse but to a lesser degree. An associated region of peak negative pressure localization was observed inside the aryepiglottic fold, which raises the question of whether the collapse or the negative pressure occurs first. In these regions of high resultant negative pressures, airway compliance will determine whether collapse occurs.

An increased amount of turbulence was observed in the rostral portions of the LLP and LLPCOR procedures for these larynges downstream from the ipsilateral aryepiglottic folds. From a clinical standpoint, these patients would receive additional revisional procedures to address this collapse, most likely resection of the aryepiglottic fold on the left side (29). It is also important to note that these larynges were isolated and this level of collapse may not have been observed in the presence of a more anatomically correct pharynx to larynx transition. The importance of the entry of airflow was established in a previous study, and by extension more upstream effects within an anatomically correct geometry are anticipated (27).

The qualitative observations of this study suggest a number of points regarding clinical upper airway surgery. The presence of irregularity in luminal tissue was associated with downstream increased turbulent kinetic energy along with negative pressure development near the wall which could then collapse. In general, the LLP and LLPCOR consistently generated the lowest impedance, and showed the most uniform distributions of pressure and velocity whereas procedures creating the more obstructive conformations generated highly variable pressure and velocity. This finding suggests that surgery should aim to maintain a funnel-like transitional entrance into the proximal trachea to create the most efficient flow.

Collapse that results in an obstruction perpendicular to the main axis of flow should be addressed surgically. It results in recirculation and negative pressure at the downstream wall which encourages further collapse of the affected tissue into the airway. This was exemplified in all larynges during the PA trial in varying degrees (Figure 10). There have been reports of horses that have presented for poor performance or noise after a PA procedure that were observed to have collapsing tissue at the surgical site (3). Increased turbulent kinetic energy is consistent with mixing and eddy formation and represents energy lost to these flow patterns and taken from effective airflow (30, 31). Higher levels of air mixing and eddy formation are strongly correlated with severity of obstruction (29, 30). This provides further evidence that revisional surgery may be worthwhile in cases with postoperative noise and poor performance as has been suggested clinically (3, 32). Another consistent feature of the PA was the presence of negative pressure and high velocity inside the left wall of the larynx. This is consistent with a report of collapse of the ipsilateral aryepiglottic fold along with other tissue structures in horses following PA (3). Interestingly, this occurs predominantly on the left side. This may be explained by the Coanda effect, in which fluid flow adheres predominantly to one side of a biconvex constriction even in potentially symmetrical situations, resulting in increased velocity and negative pressure (33). Further investigation of the role of entrance effects on these flow patterns is warranted in future studies.

The ventricular saccules contributed greatly to the flow characteristics observed in many larynges. This was most likely a function of the *ex vivo* nature of this study, but concurrent ventricular collapse has been observed in dynamic endoscopic videos of horses with RLN and following LLP (1, 2). There has been an ongoing discussion within the equine surgical community about the necessity of performing a concurrent ipsilateral ventriculocordectomy with the laryngoplasty. It has been shown to reduce the noise associated with RLN (23, 34). Additionally, it may improve postoperative laryngoplasty patients that continue to experience suboptimal performance (10, 34). The vocal fold which resides at the rostral aspect of the ventricle seems to play an undefined yet important role in swallowing, given the recent report on bulking the fold as a technique to resolve postoperative dysphagia (35). The results of this CFD study align with the clinical studies (2, 10, 34, 36) that suggest that the vocal fold and ventricle should be addressed to improve performance and noise but more research is needed to truly understand the role of these structures as they relate to both airflow and swallowing.

Limitations of this study include the basis in cadavers harvested from clinical patients, one-way crossover design, and lack of blinding between the experimental and CFD portions. While freezing cadaver larynges has been demonstrated to have minimal effects on certain biomechanical aspects of laryngeal testing (37), the effects on airflow remain unknown. Larynges were collected from the hospital population which was quite varied but necessitated by the sources available within financial and geographical reason. Additionally, randomizing the order of the surgical procedures performed would have removed handling as a confounding factor between procedures, however many efforts were made to streamline the process to reduce the amount of handling of each larynx and to minimize the time under airflow associated with each test. Additionally, this strengthened the comparison by reducing the inter-larynx variability. Further study is need to establish the relationship between the laryngeal box model and airflow in actual equine patients, despite its increasing employment in the equine airway literature. Additionally, having greater numbers of subjects would be ideal, however the time associated with processing each larynx, CT, and subsequent CFD model is significant. The lack of blinding of the primary author to the fluid mechanics portion of the study is also a limitation however the experimental and CFD phases of the study were performed approximately 2 years apart, and only the pressure data was used to run the simulations until they were finalized, then the impedances compared. True blinding to the procedures was impossible given the recognizable change in laryngeal geometry associated with each procedure. Lastly, the authors recognize the inherent bias in reporting flow characteristics from snapshot images of specific portions of the larynges as reported in the qualitative portion of this study. A combination of subject matter expertise in equine anatomy, previously published equine airway mechanics, and fluid mechanics were incorporated into the interpretation of the images.

The CFD model showed a strong agreement with the laryngeal box system for comparing the relative efficacy of the equine laryngeal surgeries reported in this study. While CFD model development should continue for numerical accuracy, the realizable κ-ε turbulence model was able to distinguish relative impedance between procedures for larynges in the majority of cases, demonstrating possible usefulness in clinical applications. Additionally, insights into the important interaction of laryngeal geometry and airflow mechanics was further delineated.

## Data availability statement

The original contributions presented in the study are included in the article/supplementary material, further inquiries can be directed to the corresponding author.

## Ethics statement

Ethical review and approval was not required for the animal study because cadaveric tissues were used, approval by the university ACUC was not needed. Written informed consent was obtained from the owners for the participation of their animals in this study.

## Author contributions

MT: first and primary author, conceptualization, data curation, formal analysis, funding acquisition, investigation, methodology, validation, visualization, and all stages of writing. DB and DW: conceptualization, funding acquisition, methodology, supervision, and writing—review and editing. JC: conceptualization, funding acquisition, methodology, supervision, and writing—review and editing, senior author. All authors contributed to the article and approved the submitted version.
